# Phytochemical Analysis and Profiling of Antioxidants and Anticancer Compounds from *Tephrosia purpurea* (L.) subsp. *apollinea* Family Fabaceae

**DOI:** 10.3390/molecules28093939

**Published:** 2023-05-07

**Authors:** Ahmed M. M. Youssef, Doaa A. M. Maaty, Yousef M. Al-Saraireh

**Affiliations:** 1 Department of Pharmacology, Faculty of Pharmacy, Mutah University, P.O. Box 7, Al-Karak 61710, Jordan; 2 Department of Botany and Microbiology, Faculty of Science, Al-Azhar University, Girls Branch, Cairo 11754, Egypt; 3 Department of Pharmacology, Faculty of Medicine, Mutah University, P.O. Box 7, Al-Karak 61710, Jordan

**Keywords:** cancers, cytotoxicity, *T. purpurea* subsp. *apollinea*, phytochemistry

## Abstract

*Tephorosia purpurea* subsp. *apollinea* was extracted with methanol and n-hexane to obtain sub-fractions. The chemical compounds identified with GC-MS and HPLC in *T. purpurea* subsp. *apollinea* extracts showed antioxidant and anticancer properties. The antioxidant and anticancer activities were investigated using DDPH and ABTS assays, and MTT assay, respectively. Stigmasta-5,24(28)-dien-3-ol, (3 β,24Z)-, 9,12,15-octadecatrienoic acid methyl ester, phytol, chlorogenic acid, and quercetin were the major chemical compounds detected in *T. purpurea* subsp. *apollinea.* These compounds possessed antioxidant and anticancer properties. The methanol extract showed antioxidant properties with DDPH and ABTS radical scavenging of 84% and 94%, respectively, relative to ascorbic acid and trolox. The anticancer effects of *T. purpurea* subsp. *apollinea* against the cancer cell lines MCF7 (IC_50_ = 102.8 ± 0.6 μg/mL), MG63 (IC_50_ = 118.3 ± 2.5 μg/mL), T47D (IC_50_ = 114.7 ± 1.0 μg/mL), HeLa (IC_50_ = 196.3 ± 2.3 μg/mL), and PC3 (IC_50_ = 117.7 ± 1.1 μg/mL) were greater than its anticancer effects against U379 (IC_50_ = 248.4 ± 7.5 μg/mL). However, it had no adverse effects on the normal cells (WI38) (IC_50_ = 242.9 ± 1.8 μg/mL). Therefore, the major active constituents presented in *T. purpurea* subsp. *apollinea* can be isolated and studied for their potential antioxidant and anticancer effects against breast, cervical, and prostate cancers and osteosarcoma.

## 1. Introduction

Despite the advancements in different therapeutic strategies, cancer continues to be a foremost cause of mortality worldwide. Conventional cancer therapies include radiotherapy, chemotherapy, and surgery. However, serious side effects and drug resistance to these treatments decrease their effectiveness [1]. The development of such problems, particularly chemotherapy drug resistance, is considered one of the main contributors to cancer-related mortality. Different multiple mechanisms are employed by cancer cells to decrease the therapeutic benefits of anticancer drugs. These include, but are not limited to, alteration in expressions of targeted proteins, DNA repair mechanisms, drug detoxification, and inactivation of medication by several catalytic proteins [2,3,4]. Therefore, there is a need to identify novel drug targets, as well as use of unique sources of anticancer drugs such as natural products [5,6]. As anticancer agents, natural products constitute an acceptable therapeutic strategy due to their availability, applicability, and low cytotoxicity. Importantly, they may provide new therapeutic strategies for combating drug resistance seen with traditional chemotherapy via several mechanisms of action.

Numerous secondary metabolites with different chemical structural diversities and biological effects have demonstrated significant potential in the treatment of various carcinomas [7]. Overall, natural products have the potential to be promising sources of novel anticancer drugs, making them an essential field of research.

The imbalance between the generation of oxidants and the antioxidant defense mechanism in the human body is known as oxidative stress [8]. Oxidative stress may cause cardiovascular and respiratory diseases such as coronary heart disease, hypertension, and chronic obstructive pulmonary disease [8]. It also may produce neurodegeneration-associated ailments such as Alzheimer’s and Parkinson’s diseases [8]. Additionally, oxidative stress strongly contributes to cancer development and metastasis [8]. Oxygen- and nitrogen-derived free radicals are reactive entities that are involved in the induction of oxidative stress that causes cellular damage [9]. Antioxidants from natural products have been widely investigated for preventing oxidative stress [10] and for the treatment of different illnesses, e.g., inflammation, carcinoma, diabetes, and neurodegenerative diseases [11]. For example, it has been demonstrated that *Portulaca oleracea* extract produced potential protective effects against neuroinflammatory disease, memory loss, and oxidative stress induced by lipopolysaccharide in mice [12].

*Tephrosia* belongs to the family Fabaceae [13]. It is widespread in tropical and subtropical regions of the world [14]. It has been widely used in folk medicine [14]. *Tephrosia purpurea* (L.) is a member of genus *Tephrosia* [13]. It has two sub-species (subsp.), *leptostachya* (DC.) Brummitt and *apollinea* (Delile) Hosni and El-Karemy [13]. Both sub-species grow in Egypt [13]. The *T. purpurea* subsp. *apollinea* is distributed in the Nile Delta, Nile banks, and desert wadis, especially Wadi Allaqi (Nubia), Gebel Elba (southern Egypt), and Sudan [13]. Several studies have revealed that the flavonoids and phenolic components of the genus *Tephrosia* have potent pharmacological effects, including pesticidal, insecticidal, and anticancer properties, especially against the human breast cancer cell line MCF7 [15]. For example, rotenoids, terpenoids, sterols, essential oils, and fixed oils have been identified as chemical compounds in the *Tephrosia* species [16]. Moreover, the *Tephrosia* species contains a large amount of flavonoids with antioxidant and anticancer effects [16]. The objective of this study is to analyze the chemical compounds in solvent subfractions of *T. purpurea* subsp. *apollinea,* in addition to investigation of their antioxidant and anticancer effects, for the first time.

## 2. Results

### 2.1. Phytochemical Compounds

**Figure 1:**
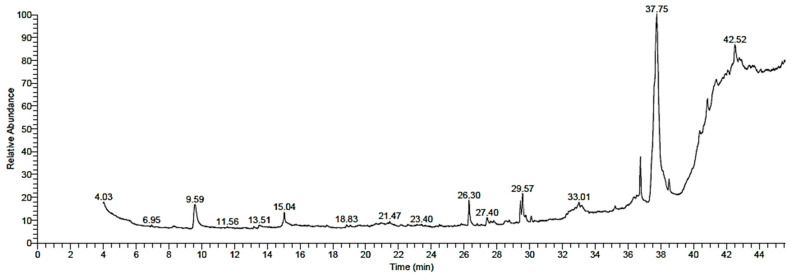
The spectra of methanol extract of *T. purpurea* subsp. *apollinea* using gas chromatography/mass spectrometry.

**Figure 2:**
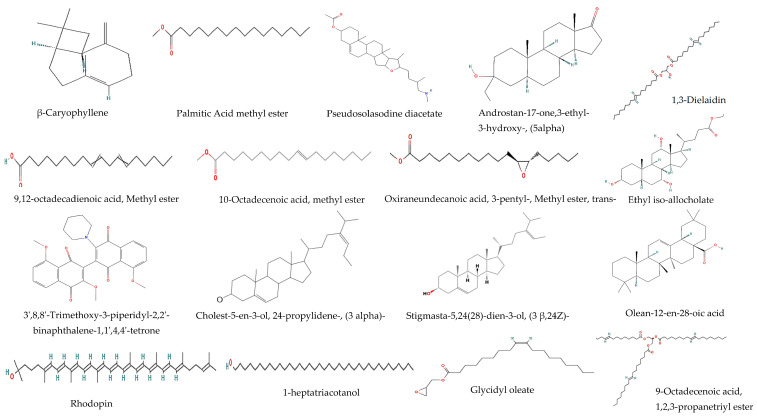
The chemical structures of the chemical compounds identified from methanol extracts from *T. purpurea* subsp. *apollinea* via GC/MS.

Many phytochemicals were present in the methanol extract of *T. purpurea* subsp. *apollinea* using GC-MS (Table 1 and Figure 1 and Figure 2). The results revealed the presence of several classes of compounds, such as steroids, triterpenoids, sesquiterpenoids, fatty acids, alkaloids, isoflavonoids, and miscellaneous compounds. The steroid stigmasta-5,24(28)-dien-3-ol,(3β,24*Z*)- relative abundance (RA = 44.74%) was the major compound in the methanol extract, followed by ethyl iso-allocholate (RA = 9.43%), a type of steroid derivative; however, androstan-17-one, 3-ethyl-3-hydroxy-, (5alpha)- (RA = 1.20%) is another steroid with small amount. The methanol extract of *T. purpurea* subsp. *apollinea* also contained two types of terpenoids, sesquiterpenoid β-caryophyllene (RA = 2.31%) and triterpenoids olean-12-en-28-oic acid (RA = 1.82%) (Figure 1 and Table 1). Fatty acid methyl esters 10-octadecenoic acid methyl ester (RA = 4.31%), palmitic acid methyl ester (RA = 4.06%), 9,12-octadecadienoic acid methyl ester (RA = 3.28%), oxiraneundecanoic acid, 3-pentyl-methyl ester, trans- (RA = 0.99%), and cholest-5-en-3-ol, 24-propylidene-, (3 alpha)- (RA = 0.65%) were found in the plant extract. Furthermore, the results revealed presence of the alkaloid pseudosolasodine diacetate (RA = 1.55%), aromatic organic compound benzene,1-methoxy-4-(1-propenyl)- (RA = 5.88%), alcoholic compound 1-heptatriacotanol (RA = 3.85%), isoflavonoid flavone 4′-OH,5-OH,7-*di*-*O*-glucoside (RA = 2.49%) and triglyceride 9-octadecenoic acid,1,2,3-propanetriyl ester (RA = 0.96%). The 3′,8,8′-Trimethoxy-3-piperidyl-2,2′-binaphthalene-1,1′,4,4′-tetrone (RA = 5.99%) is an identified oxygen organic compound and found in the methanolic extract. The carotene rhodopin (RA = 2.30%), the carboxylic ester glycidyl oleate (RA = 3.74%), and glycerol derivative 1,3-dielaidin (RA = 1.87%) were also found in the plant extract.

**Figure 3:**
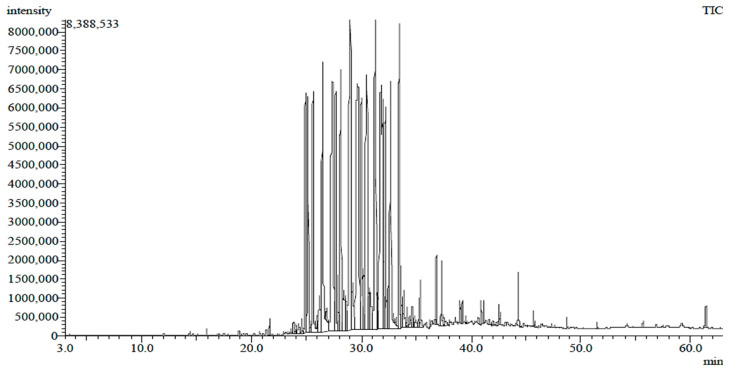
The spectra of *n*-hexane extract of *T. purpurea* subsp. *apollinea* via gas chromatography/mass spectrometry.

**Figure 4:**
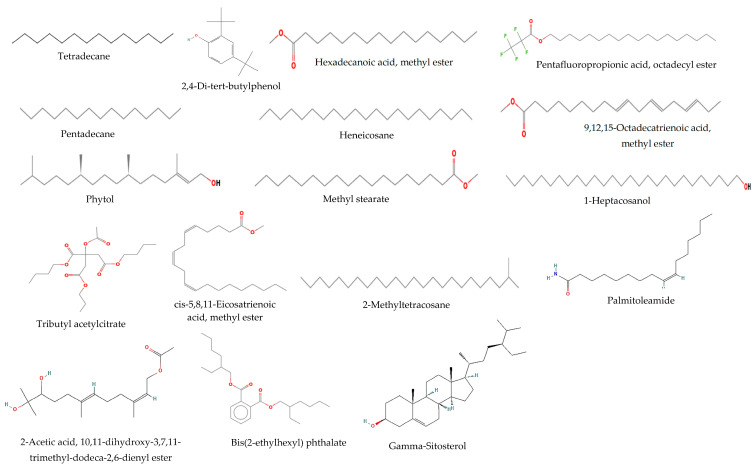
The chemical structures of the chemical compounds identified from *n*-hexan subfraction extract from *T. purpurea* subsp. *apollinea* via GC/MS.

On the other hand, it was found through GC-MS analysis that the *n*-hexane extract of *T. purpurea* subsp. *apollinea* had 17 identified components (Table 2, Figure 3 and Figure 4). These detected components were classified into esters, hydrocarbons, diterpenes, steroids, fatty acid amide, phenols, and other organic compounds. The identified fatty acid methyl esters were 9,12,15-octadecatrienoic acid methyl ester, with relative abundance (RA) of 0.67%), hexadecanoic acid methyl ester (RA = 0.37%), methyl stearate (RA = 0.07%), and cis-5,8,11-eicosatrienoic acid methyl ester (RA = 0.08%), while the identified esters were pentafluoropropionic acid octadecyl ester (RA = 0.17%), 4-ethylbenzoic acid undec-2-enyl ester (RA = 0.05%), acetic acid, 10,11-dihydroxy-3,7,11-trimethyl-dodeca-2,6-dienyl ester (RA = 0.08%), and the identified phthalate ester was a bis(2-ethylhexyl) phthalate (RA = 0.35%). The identified acyclic diterpene was a phytol (RA = 0.61%). The plant extract also contained alkanes tetradecane (RA = 0.08%), pentadecane (RA = 0.20%), 2-methyltetracosane (RA = 0.18%), and the identified aliphatic hydrocarbon was heneicosane (RA = 0.60%). The plant extract contained long-chain alcohol 1-heptacosanol (RA = 0.43%), fatty acid amide palmitoleamide (RA = 0.13%), phenol 2,4-*di*-*tert*-butylphenol (RA = 0.08%), and steroid gamma-sitosterol (RA = 0.05%). Additionally, another organic compound found in the extract was tributyl acetylcitrate (RA = 0.04%).

**Figure 5:**
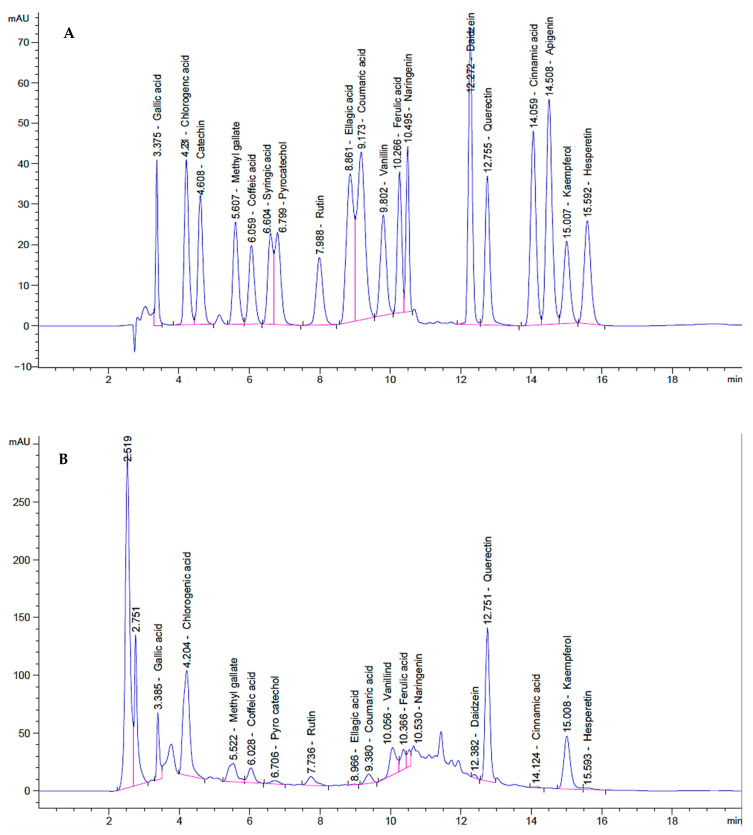
The HPLC chromatogram of methanol extract of *T. purpurea* subsp. *apollinea* (**A**) Standard chromatogram; (**B**) *T. purpurea* subsp. *apollinea* chromatogram.

Total phenolic content of aerial parts of *T. purpurea* subsp. *apollinea* was 39.12 mg GAE/g DWt, and total flavonoids amounted to 17.83 mg CE/g DWt. The content of phenolic compounds in methanolic extract of *T. purpurea* subsp. *apollinea* was determined with HPLC quantitative analysis, which led to the identification of flavonoids, flavones, flavonols, and other phenolics, as shown in Table 3 and Figure 5. The identified phenolic acids were gallic acid (1.24 mg/100 g DWt or mg%) and ferulic acid (0.14 mg/100 g DWt). Chlorogenic acid (8.10 mg/100 g DWt), pyrocatechol (0.36 mg/100 g DWt), and coumaric acid (0.15 mg/100 g DWt) were the identified phenolic compounds. The identified gallate ester was methyl gallate (0.44 mg/100 g DWt). Caffeic acid (0.66 mg/100 g DWt) was the detected polyphenol. Additionally, the phenolic aldehyde vanillin (0.45 mg/100 g DWt) was detected. The plant extract also contained the tannin ellagic acid (0.06 mg/100 g DWt) and the monocarboxylic acid cinnamate (0.01 mg/100 g DWt). Quercetin (6.76 mg/100 g DWt) and rutin (0.76 mg/100 g DWt) were the main identified flavonoids. The plant also contained the flavonol kaempferol (2.29 mg/100 g DWt), and flavanone naringenin (0.12 mg/100 g DWt), as well as hesperetin (0.06 mg/100 g DWt). The identified isoflavone was daidzein (0.07 mg/100 g DWt), while the identified flavone was apigenin (0.01 mg/100 g DWt).

### 2.2. Antioxidants Capacity

The antioxidant activity of *T. purpurea* subsp. *apollinea* methanolic extract was investigated using DPPH and ABTS radical scavenging assays (Table 4 and Table 5).

**Figure 6:**
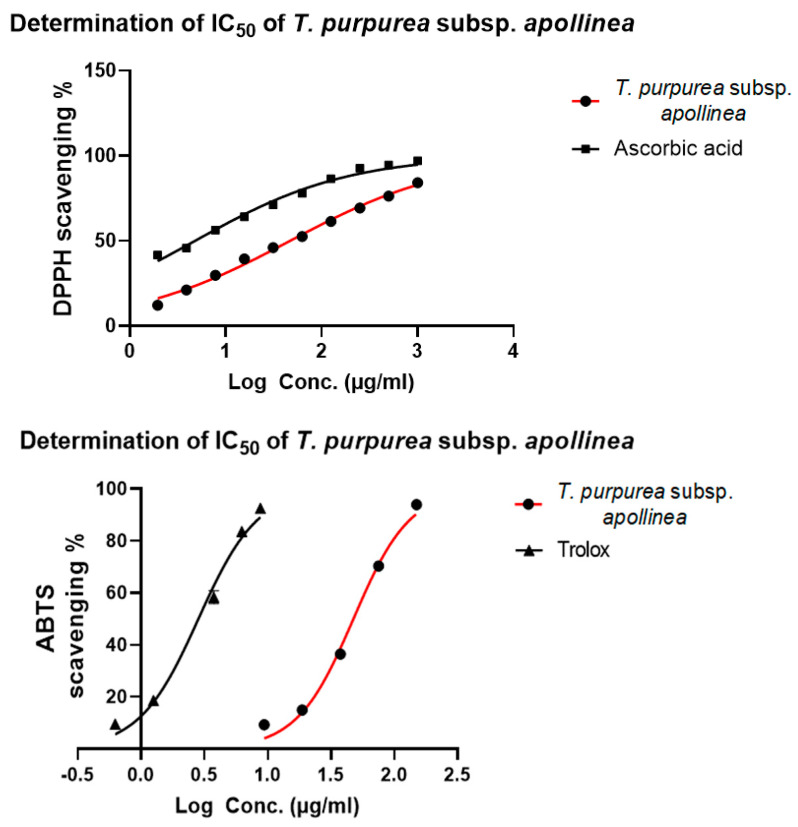
Determination of the half-maximal inhibitory concentration (IC_50_) of DPPH and ABTS radical scavenging activities of methanolic extract of *T. purpurea* subsp. *apollinea,* ascorbic acid, and trolox.

A curve was plotted showing the percentage of DPPH and ABTS radical scavenging activities vs. concentration, and the concentration of the sample required to scavenge 50% of DPPH and ABTS free radicals, which is known as IC_50_, was determined using GraphPad Prism 8 (Figure 6). The result of the DPPH assay showed that the methanol extract of *T. purpurea* subsp. *apollinea* had IC_50_ of 46.7 ± 0.7 μg/mL. However, ascorbic acid (a positive control) had IC_50_ of 4.8 ± 0.1 μg/mL. At a concentration of 1000 μg/mL of *T. purpurea* subsp. *apollinea* methanolic extract, the DPPH scavenging percentage was 84.17% (Table 4). Additionally, data from ABTS studies were comparable to the results of DPPH radical assay, and the antioxidant effect of *T. purpurea* subsp. *apollinea* methanolic extract had IC_50_ of 46.7 ± 2.6 μg/mL. However, trolox (a positive control) had IC_50_ of 2.9 ± 0.1 μg/mL. At a concentration of 150 μg/mL of *T. purpurea* subsp. *apollinea* methanolic extract, the ABTS scavenging percentage was 94.56% (Table 5). This may indicate potential antioxidant properties of *T. purpurea* subsp. *apollinea*.

### 2.3. Antitumor Capacity

**Figure 7:**
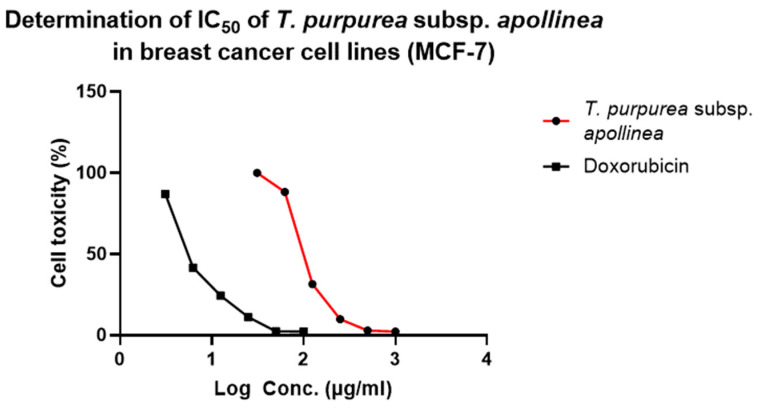
Determination of the half-maximal inhibitory concentration (IC_50_) of *T. purpurea* subsp. *apollinea* extract against breast cancer (MCF-7).

The half-maximal inhibitory concentration (IC_50_) was determined by plotting the logarithmic concentration of *T. purpurea* subsp. *apollinea* extract on the *X*-axis, and % cytotoxicity on the *Y*-axis. For example, *T. purpurea* subsp. *apollinea* showed IC_50_ of 102.85 ± 0.58 μg/mL against MCF-7, while doxorubicin, a positive control, showed IC_50_ of 7.43 ± 0.11 μg/mL (Figure 7).

**Figure 8:**
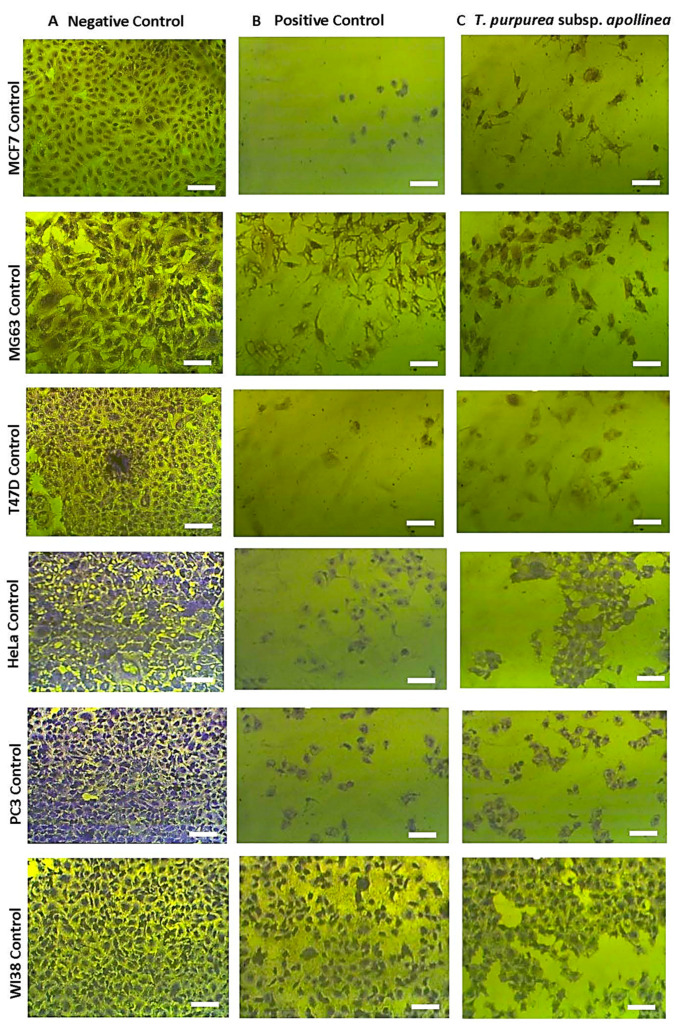
Anticancer effects of *T. purpurea* subsp. *apollinea* methanolic extract on cancer cell lines. (**A**) Complete monolayer sheets are seen in all cancer cell lines that have not been treated; (**B**) Doxorubicin (250 µg/mL) treatment results in rounded and shrunk cells in all cancer cell lines; (**C**) Shrunk cells are observed in MCF7, MG63, T47D, HeLa, and PC3 cell lines treated with *T. purpurea* subsp. *apollinea* (250 µg/mL); however, small morphological changes in WI38 are observed. The scale bar = 100 µm.

**Figure 9:**
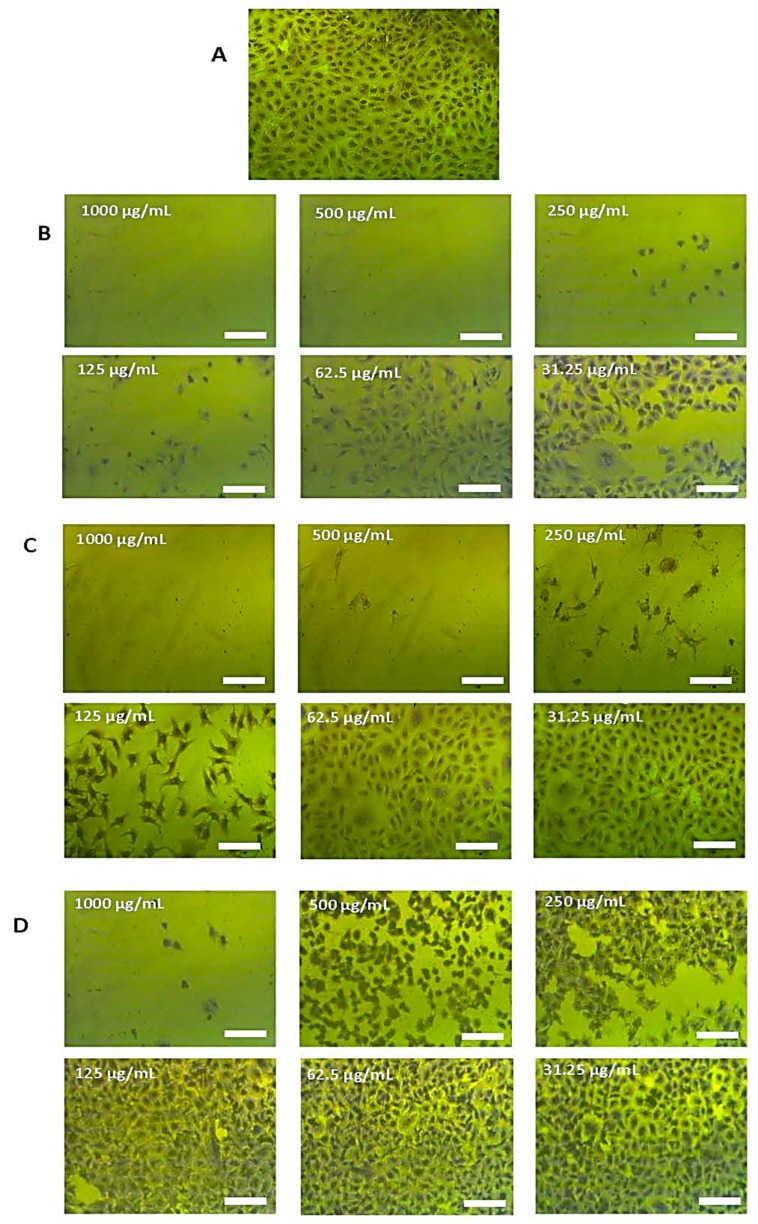
An example of anticancer effect of *T. purpurea* subsp. *apollinea* methanolic extract on breast cancer cell line (MCF7). (**A**) Complete monolayer sheets of breast cancer cells (MCF7) that have not been treated; (**B**) The effect of doxorubicin treatment at different concentrations; (**C**) The effect of *T. purpurea* subsp. *apollinea* extract on MCF7 cell lines at different concentrations; (**D**) The effect of *T. purpurea* subsp. *apollinea* extract on normal human fetal lung fibroblast (WI38). The scale bar = 100 µm.

**Figure 10:**
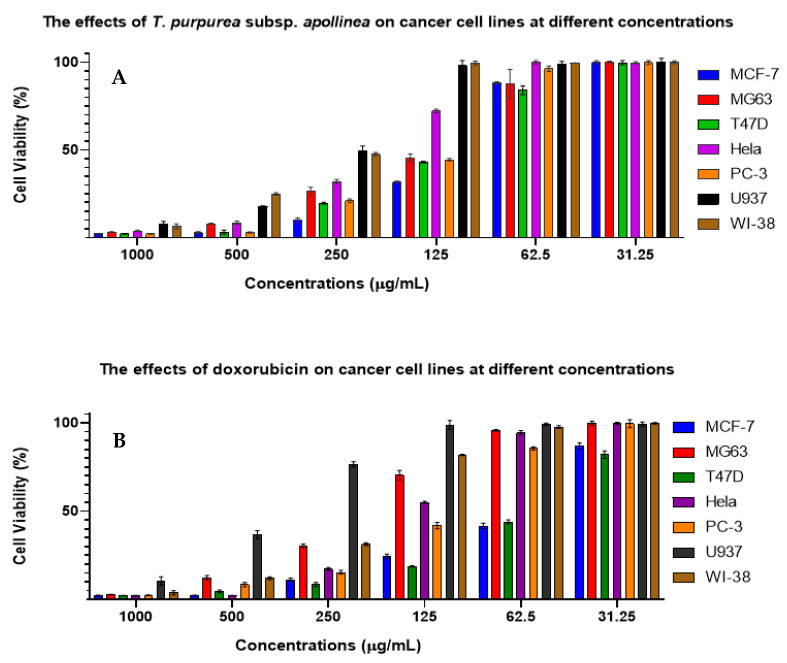
(**A**) The effects of *T. purpurea* subsp. *apollinea* methanolic extract on breast cancer (MCF7), osteosarcoma (MG63), breast ductal carcinoma (T47D), cervical cancer (HeLa), prostate cancer (PC3), leukemia (U937), and normal human fetal lung fibroblast (WI38). (**B**) The effect of doxorubicin on cancer cell lines.

The MTT assay was performed to investigate the antitumor activity of *T. purpurea* subsp. *apollinea* extract against breast cancer (MCF7), osteosarcoma (MG63), breast ductal carcinoma (T47D), cervical cancer (HeLa), prostate cancer (PC3), and leukemia (U937). Values of IC_50_ for *T. purpurea* subsp. *apollinea* extract were compared to that of doxorubicin using *t*-test analysis. Based on NCI criteria, the methanol extract of *T. purpurea* subsp. *apollinea* showed variable toxic effects (Figure 8, Figure 9 and Figure 10). The IC_50_ values for activities of *T. purpurea* subsp. *apollinea* against breast cancer (MCF7) (102.8 ± 0.5 μg/mL), osteosarcoma (MG63) (118.3 ± 2.4 μg/mL), breast ductal carcinoma (T47D) (114.7 ± 1.0 μg/mL), cervical cancer (HeLa) (196.2 ± 2.3 μg/mL), and prostate cancer (PC3) (117.6 ± 1.0 μg/mL) were mild, relative to standard drug (Table 6). In contrast, the extract activity against leukemia (U937) (248.4 ± 7.5 μg/mL) was feeble in comparison with doxorubicin. Additionally, the normal human fetal lung fibroblast (WI38) (242.9 ± 1.8 μg/mL) was weakly influenced by the cytotoxicity of *T. purpurea* subsp. *apollinea* extract, relative to doxorubicin. The SI values of *T. purpurea* subsp. *apollinea* methanol extract were calculated as indicated above. As a result, no cytotoxic selectivity for *T. purpurea* subsp. *apollinea* extract was observed, as presented in Table 7. Cells exposed to *T. purpurea* subsp. *apollinea* methanolic extract (0.2 mg/mL) for 3 days were microscopically investigated. The cell lines MCF7, MG63, T47D, HeLa, and PC3 were rounded and shrunk by the methanolic extract of *T. purpurea* subsp. *apollinea.* However, WI38 cell lines demonstrated small changes in morphology when treated with *T. purpurea* subsp. *apollinea*, compared to their control cell lines (Figure 8 and Figure 9). The U937 cell lines could not be microscopically examined.

## 3. Discussion

The methanolic extract of *T. purpurea* subsp. *apollinea* was analyzed using GC-MS, and the identified compounds were shown to possess antioxidant and anticancer properties. These compounds were responsible for the antioxidant and anticancer effects of *T. purpurea* subsp. *apollinea* on most of the tested cancer cell lines. The identified sesquiterpenoid that has been associated with antioxidant and anticancer properties was β-caryophyllene [17]. The palmitic acid methyl ester [18,19], 9,12-octadecadienoic acid methyl ester [22], 10-octadecenoic acid methyl ester [23], and oxiraneundecanoic acid 3-pentyl- methyl ester, trans [24] are fatty acid methyl esters that exert antioxidant and anticancer effects. Pseudosolasodine diacetate has been reported as an alkaloid compound with antioxidant properties [20]. Additionally, the identified steroid was ethyl iso-allocholate which has been reported to possess antioxidant properties [25]. The other identified steroid, stigmasta-5,24(28)-dien-3-ol, (3β,24*Z*), which was present at the highest level among the compounds in the plant, has been reported as an antioxidant [28]. Androstan-17-one, 3-ethyl-3-hydroxy-,(5alpha) is also an identified steroid with antioxidant properties [21]. The detected isoflavonoid was a flavone 4’-OH,5-OH,7-*di*-*O*-glucoside with antioxidant and anticancer properties [29,30]. Additionally, 1-heptatriacotanol is an identified alcoholic compound and is reported to have anticancer properties [32]. The oxygen organic compound 3’,8,8’-trimethoxy-3-piperidyl-2,2’-binaphthalene-1,1’,4,4’-tetrone has been reported with antioxidant and antibacterial properties [26]. Rhodopin is an identified carotene and has been reported to have antioxidant properties [31]. The carboxylic ester which has been reported with anticancer properties is glycidyl oleate [33]. Other identified compounds have not been evaluated for either antioxidant or anticancer properties. However, they have been reported with other biological activities. These are the fatty acid cholest-5-en-3-ol, 24-propylidene-(3 alpha) [27], and the triglyceride 9-octadecenoic acid, 1,2,3-propanetriyl ester [34,35] that have been reported with antibacterial and inflammation suppressing effects, respectively. However, the biological properties of the identified triterpenoid olean-12-en-28-oic acid, the aromatic organic compound benzene,1-methoxy-4-(1-propenyl), and glycerol derivative 1,3-dielaidin have not been evaluated until now.

The identification of the compounds of n-hexane subfraction extract of *T. purpurea* subsp. *apollinea* was performed with GC-MS. The majority of compounds in n-hexane subfraction extract possess antioxidant and anticancer properties. The identified phenol with antioxidant activity was 2,4-*di*-*tert*-butylphenol [37]. Additionally, hexadecanoic acid methyl ester [39], 9,12,15-octadecatrienoic acid methyl ester, which was present at the highest level relative to the other constituents [42], and methyl stearate [45] were the identified fatty acids methyl esters that are known to exert antioxidant and anticancer effects. However, the fatty acid methyl ester cis-5,8,11-eicosatrienoic acid methyl ester exerts anti-inflammation-related effects [48]. The identified diterpene was phytol, of which there was a considerable amount relative to the other components, and it has antioxidative stress and anticancer activities [43,44]. The long-chain alcohol 1-heptacosanol has been reported to be an antioxidant [46]. The identified fatty acid amide with antioxidant properties was palmitoleamide [50]. Bis(2-ethylhexyl) phthalate exerts antioxidant and antitumor effects [52]. Additionally, gamma-sitosterol is a steroid that has been reported to possess anticancer activity [54]. The other identified compounds have not been evaluated for either antioxidant or anticancer properties; however, they have been evaluated for different biological activities. For example, pentadecane [38], tetradecane [36], and 2-methyltetracosane [49] are alkanes with antibacterial effects. An aliphatic hydrocarbon (heneicosane) [41] and carbonyl compound (tributyl acetylcitrate) [47] have been reported to possess pesticidal and antimicrobial activities, respectively, while acetic acid, 10,11-dihydroxy-3,7,11-trimethyl-dodeca-2,6-dienyl ester [51] and pentafluoropropionic acid octadecyl ester [40] are the identified esters and have been reported with insecticidal and antimicrobial properties, respectively.

The phenolic acids and flavonoids found in methanolic *T. purpurea* subsp. *apollinea* extract may have the potential to actively participate in the antioxidant and anticancer effects on the tested carcinoma cells. These phytochemical components were identified with the HPLC apparatus, which analysis showed the presence of phenolic acid (gallic acid), which has been reported to have antioxidant and anticancer activities [55,56]. Another phenolic acid identified in *T. purpurea* subsp. *apollinea* methanolic extract was ferulic acid, which has also been reported to have antioxidant and anticancer activities [65]. Chlorogenic acid [57], pyrocatechol [60], and coumaric acid [63] were the identified phenolic compounds in *T. purpurea* subsp. *apollinea* extract with antioxidant and anticancer properties. The identified gallate ester in the extract (methyl gallate) has also been reported to have antioxidant and anticancer activities [58]. Additionally, caffeic acid was the identified polyphenol, and it is known to exert antioxidative stress and anticancer activities [59]. The tannin (ellagic acid) also has antioxidant and anticancer potential [62]. The flavonoids rutin [61] and quercetin [68] have been reported to have antioxidant and anticancer properties. Additionally, naringenin [66] and hesperetin [72] were the identified flavanones known to have antioxidant and anticancer properties. These findings agree with the results reported in the literature where the flavanones (-)-pseudosemiglabrin isolated from *T. apollinea,* which has a close affiliation to *T. purpurea* subsp. *apollinea* showed anticancer effects against leukemia, breast, and prostate cancers [73]. Daidzein is an isoflavone that has also been reported to exert antioxidant and anticancer properties [67]. Cinnamic acid is monocarboxylic acid present in *T. purpurea* subsp. *apollinea,* and studies have shown that it has antioxidant and anticancer properties [69]. The phenolic aldehyde found in the extract with reported antioxidant and anticancer properties was vanillin [64]. Apigenin [70] and kaempferol [71] were the identified flavone and flavonol, respectively, and have been reported to have antioxidant and anticancer properties. These results agree with existing data on different extracts from *T. apollina,* which contained flavones (semiglabrin 1, pseudosemiglabrin 2, glabratephrin 3, and apollinine 4) [74]. Interestingly, these extracts showed various degrees of antioxidant and anticancer effects against hepatocellular carcinoma (HepG2), colorectal carcinoma (HC116), and prostate cancer (PC3) [74]. Additionally, another prenylated flavone (isoglabratephrin) isolated from *T. apollinea* showed anticancer activities against prostate cancer (PC3) and pancreatic cancer (PANC1) through induction of chromatin disruption and nuclear damage [75]. This is consistent with the anticancer effects of *T. purpurea* subsp. *apollinea* against breast cancer (MCF7), ductal breast cancer (T47D), osteosarcoma (MG63), cervical cancer (HeLa), and prostate cancer, as seen in this research. However, the anticancer effect was weak against leukemia, when compared with other cancer cell lines. Therefore, *T. purpurea* subsp. *apollinea* exerted anticancer impact on all cells investigated, except leukemia. However, the anticancer effects of *T. purpurea* subsp. *apollinea* were without any selective cytotoxicity to cancer cells, relative to non-cancer cell lines (WI38). In this study, *T. purpurea* subsp. *apollinea* methanolic extract also demonstrated potential dose-dependent antioxidant activities, when compared to ascorbic acid. This result is consistent with the findings of dose-dependent inhibition of DPPH radical by *T. apollinea* methanolic extract [76]. Additionally, different extracts of *T. apollinea* have been reported to have antioxidant activities when assessed for radical scavenging, TAC, anti-lipid peroxidation, and GSH level [77]. Therefore, *T. purpurea* subsp. *apollinea* may have potential antioxidant properties due to the various identified antioxidant compounds, and it may also have anticancer properties due to the presence of different anticancer compounds.

## 4. Materials and Methods

### 4.1. Plant Material

The aerial parts of *T. purpurea* subsp. *apollinea* were collected from the Al-Mansoura city (31°02′27.2″ N 31°22′42.6″ E), Delta region, Egypt, at the flowering stage in March 2022, and the voucher sample was kept at its herbarium (CAIH-21/23-5) in Cairo, Egypt after it was authenticated by a plant taxonomist Professor Iman Hussein Salama Al-Gohary. Following rinsing in running water and shade dehydration for 1 week and 3 days at 25 °C, the specimen was ground to powder [7].

### 4.2. Preparation of Methanolic Extract and GC-MS Studies

Utilizing the cold percolation method, 200 g of the above sample of the plant was subjected to extraction. Subsequently, the extract was exposed to three separate applications of 500 mL of 70% methanol for 72 h at 25 °C. The methanol extract was filtered using a Buchner funnel. Then, the remaining methanol was entirely removed from the methanol extract using a rotary evaporator and concentration at low pressure at 40 °C. The sediment was dried in a desiccator to produce a dry weight yield of 20.68 g/100 g of *T. purpurea* subsp. *apollinea,* and GC-MS analysis was employed to identify the bioactive components [7].

A TRACE 1310 gas chromatograph connected to an ISQLT MS single quadrupole mass spectrometer (Thermo Fisher Scientific, Waltham, MA, USA) was used. The data were obtained from the GC-MS at 70 eV ionization voltage, EI ionization mode, DB5-MS column with an internal diameter of 0.25 mm (J & W Scientific, Folsom, CA, USA), and the temperature was programmed in this manner: 3 min at 40 °C, 5 min at 280 °C, 1 min at 290 °C and constant at 7.5 °C/min. The detector and injector temperatures were adjusted to be 300 and 200 °C, respectively. The flow rate of the carrying gas (helium) was constant at 1 mL/min. The WILEY and NIST Mass Spectral Data Base was used as a search library [7].

### 4.3. Sub-Fractionation Using n-Hexane and GC-MS Studies

In 250 mL of distilled water, 2.5 g of lyophilized crude methanolic extract of *T. purpurea* subsp. *apollinea* was re-dissolved. Then, for 24 h, the re-dissolved crude extract was separated with n-hexane using a separatory funnel, followed by the use of a rotating evaporator at a temperature of 40 °C and lower pressures to obtain the *n*-hexane layer in a dry form. A GC-MS analysis was performed on the dried portion (1 mg) [78,79].

A split-splitless injector was used in tandem with the Shimadzu GCMS-QP2010 (Shimadzu, Tokyo, Japan). The mass spectra were acquired using a Restek 30-m Rtx-5MS column with an internal diameter of 0.25 mm (Chrom Tech, Bellefonte, PA, USA). The starting temperature of the column was raised to 300 °C at a rate of 5 °C/min for 5 min and was then held constant there for 2 min (isothermal). The injector temperature was 250 °C. Helium carrier gas was at a flow rate of 1.41 mL per min. The ion source of 200 °C, ionization voltage of 70 eV, and filament emission current of 60 mA were applied to acquire all mass spectra. The sample (1% *v*/*v*) dilution injection was performed via a split mode [78].

### 4.4. Quantification of Phenolics and Flavonoids

Phenolics and flavonoids were quantified using the standard Folin–Ciocalteu technique [79,80]. The optical density of the bluish reaction solution was read at a maximum λ_max_ of 725 nm after one hour using the Unicam UV-visible Spectrometer, with distilled H_2_O as blank. A calibration curve of gallic acid was plotted. The results were calculated in terms of milligrams of gallic acid equivalents (GAE) per gram dry weight [79,80].

The total flavonoid content was determined using an aluminum chloride colorimetric method [79,80]. The extract was diluted 1:6 (v:v) with distilled water, and the mixture was then added to 75 µL of NaNO_2_ (5%), followed by the addition of 10% AlCl_3_.6H_2_O (150 µL) after 6 min to the mixture and it was let to stand for an additional 5 min. After adding 1 M NaOH solution (0.5 mL), 2.5 mL of distilled water was added to the mixture. Optical density was recorded at 510 nm against a blank of distilled water. A standard calibration curve was developed using (+)-catechin. The findings were calculated as mg of catechin equivalents (CE) for each gram [79,80].

### 4.5. HPLC Analysis of Phenolic Compounds

#### 4.5.1. Standards

The phenolic compounds were investigated with HPLC reagents (acetonitrile, methanol, and trifluoroacetic acid) purchased from SDS (Peypin, France). The distilled water was obtained from Milli-Q (Millipore, MA, USA). All standards used (methyl gallate, coffeic acid, etc.) were provided by Sigma (St. Louis, MO, USA) and had a 98% level of purity [79,80].

#### 4.5.2. HPLC Quantitation of Phenolics

The MeOH extract of *T. purpurea* subsp. *apollinea* (0.20 g) was solubilized in 2 mL of acetonitrile. The identification of phenolic compounds was performed using an Agilent 1260 HPLC instrument (Agilent Technologies, Santa Clara, CA, USA). An Eclipse C18 column (4.6 mm × 250 mm i.d., 5 μm) was used to separate the phenolic compounds, with a mobile phase comprised of H_2_O (A) and CF_3_COOH (0.05%) in acetonitrile (B) at a flow rate of 0.9 mL/60 s.

A linear gradient was applied to configure the mobile phase. Sample monitoring was performed at 280 nm using a multiple λ_max_ detector. For each sample solution, 5 µL was injected into the column at 40 °C. Standards were prepared as stock solutions of 10 mg/50 mL in methanol. Then, the standards were loaded into HPLC after being diluted. The flavonoids and phenolic acids in the methanolic extract from *T. purpurea* subsp. *apollinea* were identified and quantified using Equation (1), and the results were calculated in terms of mg/100 g dry weight [79,80].

(1) $$\;Conc.\;of\;the\;identified\;compound\;\left(\mu g/\mathrm{mL}\right)\;=\frac{Area\;of\;the\;sample\times Conc.\;\;\left(\mu g/\mathrm{mL}\right)of\;the\;standard\;}{Area\;of\;the\;standard}\;\;\;\;\;\;\;\;\;\;\;\;\;\;\;\;\;\;\;\;\;$$

### 4.6. Evaluation of Antioxidant Properties

#### 4.6.1. DPPH Antioxidant Assay

The 2,2-diphenylpicrylhydrazyl (DPPH) neutralizing potential of methanol extract of *T. purpurea* subsp. *apollinea* was investigated. The methanolic extract of *T. purpurea* subsp. *apollinea* was serially diluted to various concentrations, i.e., 1.95, 3.9, 7.8, 15.6, 31.2, 62.5, 125, 250, 500, and 1000 µg/mL. Each methanolic extract concentration (1 mL) was added to 3 mL of 0.1 mM DPPH solubilized in methanol, followed by shaking and placing it into a dark chamber for 30 min. When DPPH reacts with an H-donating antioxidant, it is scavenged, thereby resulting in a decrease in absorbance [80]. The optical density of each concentration was measured at 517 nm in a UV-Vis spectrophotometer. Ascorbic acid was used as an antioxidant standard. All the values were measured in triplicate.

#### 4.6.2. ABTS Antioxidant Assay

Distilled water was used to bring up a 50-mL volumetric flask to mark after dissolving 192 mg of 2,2′-azino-bis(3-ethylbenzothiazoline-6-sulfonic acid) (ABTS) in a small volume of water and transferring it to the flask, followed by addition of 1 mL of this solution to 17 μL 0.14 M K_2_S_2_O_8_, and incubation in darkness for 24 h. To prepare the final ABTS dilution for the *T. purpurea* subsp. *apollinea* extract, 1 mL of reaction mixture was added to 49 mL of methanol. Then, 0.190 mL of newly made ABTS solution was mixed with 0.010 mL of plant extract in a 96-well plate and kept in the dark for 30 min. Thereafter, a decrease in ABTS OD was read at 734 nm in a FluoStar Omega microplate reader. Trolox was used as an antioxidant standard. All the values were measured in triplicate.

#### 4.6.3. Measurement of IC50

The IC50 values of DPPH and ABTS antioxidant assays for samples and controls were calculated using GraphPad Prism 7. The IC_50_ values were calculated as shown in Equation (2):

(2) Inhibition percent=Average absorbance of blank−Average absorbance of test×100    

### 4.7. Determination of Anticancer Effect

Breast cancer cell lines (MCF7), osteosarcoma (MG63), breast ductal carcinoma (T47D), leukemia (U937), as well as HeLa, PC3, and healthy pulmonary fibroblast were supplied by the tissue culture laboratory at Vacsera, Egypt. The culturing procedure was maintained sterile, utilizing a laminar airflow cabinet. The cells were cultured in Roswell Park Memorial Institute medium (RPMI 1640). The medium was provided with antibiotics (streptomycin and penicillin) as well as antifungal agents (amphotericin B) and l-glutamine. It was also supplemented with 10% heat-inactivated fetal bovine serum [80].

#### 4.7.1. Viability Evaluation

Cell viability was measured using a 3-(4,5-dimethylthiazol-2-yl)-2,5-diphenyl-2H-tetrazolium bromide (MTT) assay. The principle of the MTT assay is that through mitochondrial reduction, purple-colored crystals are produced from the yellowish MTT [80]. A 96-well microplate was used for inoculation, and 100 µL of the Roswell Park Memorial Institute medium (RPMI 1640) was added to each well. A fully formed monolayer sheet was produced by incubation of the microplate for 24 h at 5% CO_2_, 37 °C, and 95% humidity. When the cells had formed a confluent layer, the growth medium was discarded.

Using a growth medium, serial dilutions of the dimethyl sulfoxide (DMSO)-solubilized extract were produced at concentrations of 31.25, 62.5, 125, 250, 500, and 1000 µg/mL [80]. Using a multichannel pipette, the cells were transferred to 0.10 mL of each extract concentration in triplicate before being dispersed in 96-well plates, followed by incubation of the extract-treated cells for 24 h at 37 °C and 5% CO_2_. Control cells were incubated without the addition of the stem and leaf extracts. Thereafter, 20 µL of MTT solution (5 mg/mL) in PBS was added to each well, followed by mixing, which was performed for 5 min at 150 rpm. After that, incubation was maintained for 4 h. Then, the formazan crystals were taken up in 200 µL of DMSO and vigorously agitated. A microplate reader was used to measure the optical density of the formazan solution at 560 nm, with values corrected using a background reference λ of 620 nm [80]. Each experiment was performed three times.

#### 4.7.2. Measurement of IC_50_

The IC_50_ profiles of methanol extract of *T. purpurea* subsp. *apollinea* and a positive control for cancer and healthy cell lines were determined with GraphPad Prism version 7 (GraphPad Software Inc., San Diego, CA, USA). The IC_50_ values were computed as shown in Equation (3) [80]. The data obtained were subjected to non-linear regression to obtain 50% effective concentrations (EC50) and cytotoxic concentration (CC50), with 95% confidence intervals.

(3) $$\mathrm{Inhibition}\;\mathrm{percent}=\frac{100-\left(\mathrm{mean}\;\mathrm{OD}\;\mathrm{test}\right)}{\mathrm{Mean}\;\mathrm{OD}\;\mathrm{control}}\times 100\;$$

#### 4.7.3. Classification of Cytotoxicity

The United States NCI and Geran guidelines were used to classify the cytotoxicity of *T. purpurea* subsp. *apollinea* methanol extract, based on IC_50_, with IC_50_ values ≤ 20, 21–200, 201–500, and >501 µg/mL classified as extremely-, mildly-, weakly-, and non-cytotoxic, respectively [81,82].

#### 4.7.4. Criteria for Selectivity

The selectivity index (SI) is IC_50_ for a healthy cell (WI38) divided by IC_50_ for a cancerous cell. SI values less than 3 indicate non-specificity to cancer cells [81]. The SI values of methanolic extract of *T. purpurea* subsp. *apollinea* were calculated using Equation (3) as follows:

(4) $$\mathrm{SI}=\frac{{\mathrm{IC}}_{50}\;\mathrm{for}\;a\;\mathrm{healthy}\;\mathrm{cell}\;\left(\mathrm{WI}38\right)}{\begin{array}{c} {\mathrm{IC}}_{50}\;\mathrm{for}\;a\;\mathrm{cancerous}\;\mathrm{cell} \end{array}}$$

#### 4.7.5. Microscopy

Morphologies of cells treated with the various methanol *T. purpurea* subsp. *apollinea* extract concentrations were investigated under light microscopy at 10× objective lens, total magnification = 100×.

## 5. Conclusions

*Tephrosia purpurea* subsp. *apollinea* contained various chemical compounds in the methanolic and n-hexane subfraction extracts. The major components that had antioxidant and anticancer properties were stigmasta- 14 5,24(28)-dien-3-ol, (3 β,24Z)-, 9,12,15-octadecatrienoic acid methyl ester, phytol, chlorogenic acid, and quercetin. Therefore, these chemical compounds may be isolated from *T. purpurea* subsp. *apollinea* and their antioxidant and anticancer properties may be investigated against breast cancer (MCF7), osteosarcoma (MG63), breast ductal carcinoma (T47D), cervical cancer (HeLa), and prostate cancer (PC3). However, the anticancer effect of *Tephrosia purpurea* subsp. *apollinea* was weak against leukemia (U937). Additionally, the healthy cell lines (WI-38) were not greatly affected by the *Tephrosia purpurea* subsp. *apollinea* cytotoxicity. Moreover, this is the first report on this plant, and it may be the foundation for further pharmacological studies.

## Figures and Tables

**Table 1:** Biological compounds identification of methanol extract of *T. purpurea* subsp. *apollinea* by GC/MS.

	Compounds	MW	M.F.	Category	Rt	RA%	Biological Activities	References
1	Benzene, 1-methoxy-4-(1-propenyl)-	148	C_10_H_12_O	Aromatic organic compound	9.58	5.88	No data available	
2	β-Caryophyllene	204	C_15_H_24_	Sesquiterpenoid	15.04	2.31	Antioxidant and Anticancer	[17]
3	Palmitic Acid methyl ester	270	C_17_H_34_O_2_	Fatty acid methyl ester	26.30	4.06	Antioxidant and Anticancer	[18,19]
4	Pseudosolasodine diacetate	499	C_31_H_49_NO_4_	Alkaloid Compound	27.40	1.55	Antioxidant	[20]
5	Androstan-17-one, 3-ethyl-3-hydroxy-, (5alpha)	318	C_21_H_34_O_2_	Steroid	28.80	1.20	Antioxidant and Antibacterial	[21]
6	9,12-octadecadienoic acid, Methyl ester	294	C_19_H_34_O_2_	Fatty acid methyl ester	29.43	3.28	Anticancer	[22]
7	10-Octadecenoic acid, methyl ester	296	C_19_H_36_O_2_	Fatty acid methyl ester	29.57	4.31	Antioxidant	[23]
8	Oxiraneundecanoic acid, 3-pentyl-, Methyl ester, trans-	312	C_19_H_36_O_3_	Fatty acid methyl esters	30.09	0.99	Antioxidant and Anticancer	[24]
9	Ethyl iso-allocholate	436	C_26_H_44_O_5_	Steroid	33.00	9.43	Antioxidant	[25]
10	3’,8,8’-Trimethoxy-3-piperidyl-2,2’-binaphthalene-1,1’,4,4’-tetrone	487	C_28_H_25_NO_7_	Oxygen organic compound	36.75	5.99	Antioxidant and antimicrobial	[26]
11	Cholest-5-en-3-ol, 24-propylidene-, (3 alpha)-	426	C_30_H_50_O	Fatty acid	37.45	0.65	Antibacterial	[27]
12	Stigmasta-5,24(28)-dien-3-ol, (3 β,24*Z*)-	412	C_29_H_48_O	Steroid	37.75	38.74	Antioxidant	[28]
13	Olean-12-en-28-oic acid	440	C_30_H_48_O_2_	Triterpenoids	38.50	1.82	No data available	
14	Flavone 4’-OH,5-OH,7-*di*-*O*-glucoside	594	C_27_H_30_O_15_	Isoflavonoid	40.36	2.49	Antioxidant and Anticancer	[29,30]
15	Rhodopin	554	C_40_H_58_O	Carotene	40.51	2.30	Antioxidant	[31]
16	1-heptatriacotanol	537	C_37_H_76_O	Alcoholic compound	40.84	3.85	Antioxidant and Anticancer	[32]
17	Glycidyl oleate	338	C_21_H_38_O_3_	Ester	42.51	3.74	Anticancer	[33]
18	9-Octadecenoic acid, 1,2,3-propanetriyl ester	884	C_57_H_104_O_6_	Triglyceride	42.93	3.45	Antioxidant and immune modulators	[34,35]
19	1,3-Dielaidin	620	C_39_H_72_O_5_	Glycerol Derivatives	43.70	1.87	No data available	

**Table 2:** Biological compounds identification of n-hexane extract of *T. purpurea* subsp. *apollinea* using GC/MS.

	Compounds	MW	M.F.	Category	Rt	RA%	Biological Activities	References
1	Tetradecane	198	C_14_H_30_	Alkane	21.64	0.08	Antibacterial	[36]
2	2,4-*Di*-*tert*-butylphenol	206	C_14_H_22_O	Phenol	23.99	0.08	Antioxidant	[37]
3	Pentadecane	212	C_15_H_32_	Alkane	24.26	0.20	Antibacterial	[38]
4	Hexadecanoic acid, methyl ester	270	C_17_H_34_O_2_	Fatty acid methyl esters	33.62	0.37	Antioxidant	[39]
5	Pentafluoropropionic acid, octadecyl ester	416	C_21_H_37_F_5_O_2_	Ester	35.24	0.17	Antibacterial	[40]
6	Heneicosane	296	C_21_H_44_	Aliphatic hydrocarbon	35.42	0.60	Pesticidal	[41]
7	9,12,15-Octadecatrienoic acid, methyl ester	292	C_19_H_32_O_2_	Fatty acid methyl ester	36.86	0.67	Anticancer	[42]
8	Phytol	296	C_20_H_40_O	Acyclic diterpene alcohol	37.31	0.61	Antioxidant and anticancer	[43,44]
9	Methyl stearate	298	C_19_H_38_O_2_	Fatty acid methyl ester	37.52	0.07	Antioxidants and anticancer	[45]
10	1-Heptacosanol	396	C_27_H_56_O	Long-chain fatty alcohol	39.02	0.43	Antioxidant	[46]
11	Tributyl acetylcitrate	402	C_20_H_34_O_8_	Organic compound	39.54	0.04	Antimicrobial	[47]
12	*cis*-5,8,11-Eicosatrienoic acid, methyl ester	320	C_21_H_36_O_2_	Fatty acid methyl ester	40.631	0.08	Anti-inflammatory	[48]
13	2-Methyltetracosane	352	C_25_H_52_	Alkane	40.95	0.18	Antibacterial	[49]
14	Palmitoleamide	253	C_16_H_31_NO	Fatty acid amide	41.16	0.13	Antioxidant	[50]
15	Acetic acid, 10,11-dihydroxy-3,7,11-trimethyl-dodeca-2,6-dienyl ester	298	C_17_H_30_O_4_	Ester	41.65	0.08	Insecticidal	[51]
16	Bis(2-ethylhexyl) phthalate	390	C_24_H_38_O_4_	Phthalate ester	44.35	0.35	Antioxidant and Anticancer	[52,53]
17	Gamma-Sitosterol	414	C_29_H_50_O	Steroid	55.66	0.05	Anticancer	[54]

**Table 3:** Phenolic compounds identification of methanol extract of *T. purpurea* subsp. *apollinea* using HPLC.

No.	Compounds	MW	M.F.	Category	Rt	mg/100 g DWt	Biological Activities	References
1	Gallic acid	170	C_7_H_6_O_5_	Phenolic acids	3.38	1.24	Antioxidant and Anticancer	[55,56]
2	Chlorogenic acid	354	C_16_H_18_O_9_	Phenolic compound	4.20	8.10	Antioxidant and Anticancer	[57]
4	Methyl gallate	184	C_8_H_8_O_5_	Gallate ester	5.52	0.44	Antioxidant and Anticancer	[58]
5	Coffeic acid	180	C_9_H_8_O_4_	Polyphenol	6.02	0.66	Antioxidant and Anticancer	[59]
7	Pyrocatechol	110	C_6_H_6_O_2_	Phenolic compounds	6.70	0.36	Antioxidant and Anticancer	[60]
8	Rutin	610.5	C_27_H_30_O_16_	Flavonoid	7.73	0.76	Antioxidant and Anticancer	[61]
9	Ellagic acid	302	C_14_H_6_O_8_	Tannins	8.96	0.06	Antioxidant and Anticancer	[62]
10	Coumaric acid	164	C_9_H_8_O_3_	Phenolic compound	9.38	0.15	Antioxidant and Anticancer	[63]
11	Vanillin	152	C_8_H_8_O_3_	Phenolic aldehyde	10.05	0.45	Antioxidant and Anticancer	[64]
12	Ferulic acid	194	C_10_H_10_O_4_	Phenolic acid	10.36	0.14	Antioxidant and Anticancer	[65]
13	Naringenin	580.5	C_27_H_32_O_14_	Flavanones	10.53	0.12	Antioxidant and Anticancer	[66]
14	Daidzein	254	C_15_H_10_O_4_	Isoflavone	12.38	0.07	Antioxidant and Anticancer	[67]
15	Quercetin	302	C_15_H_10_O_7_	Flavonoid	12.75	6.76	Antioxidant and Anticancer	[68]
16	Cinnamic acid	148	C_9_H_8_O_2_	Monocarboxylic acid	14.12	0.01	Antioxidant and Anticancer	[69]
17	Apigenin	270	C_15_H_10_O_5_	Flavones	14.50	0.01	Antioxidant and Anticancer	[70]
18	Kaempferol	286	C_15_H_10_O_6_	Flavonol	15.00	2.29	Antioxidant and Anticancer	[71]
19	Hesperetin	302	C_16_H_14_O_6_	Flavanone	15.59	0.06	Antioxidant and Anticancer	[72]

**Table 4:** DPPH radical scavenging of *T. purpurea* subsp. *apollinea* extracts and ascorbic acid.

	DPPH Scavenging%			
Conc. µg/mL	*T. purpurea* subsp. *apollinea*		Ascorbic Acid	
1000	84.1 ± 0.3 ***	^a^ IC_50_ = 46.7 ± 0.7 *** μg/mL	97.1 ± 0.1	^a^ IC_50_ = 4.8 ± 0.1 μg/mL
500	76.2 ± 0.1 ***		94.5 ± 0.2	
250	69.3 ± 0.3 ***		92.7 ± 0.2	
125	61.4 ± 0.1 ***		86.4 ± 0.3	
62.5	52.6 ± 0.1 ***		77.9 ± 0.3	
31.25	46.0 ± 0.2 ***		71.2 ± 0.2	
15.625	39.4 ± 0.2 ***		64.2 ± 0.3	
7.8125	29.8 ± 0.6 ***		56.2 ± 0.2	
3.9	21.2 ± 0.6 ***		45.9 ± 0.1	
1.95	12.2 ± 0.2 ***		41.8 ± 0.5	

^a^ IC_50_: The half-maximal inhibitory concentration. The findings are represented as mean ± standard deviation. *** *p* = 0.0001 show significant changes in comparison to ascorbic acid. The *T. purpurea* subsp. *apollinea* methanolic extract and ascorbic acid were compared using a *t*-test.

**Table 5:** ABTS radical scavenging of *T. purpurea* subsp. *apollinea* extracts and trolox.

	ABTS Scavenging%				
Conc. µg/mL	*T. purpurea* subsp. *apollinea*		Conc. µg/mL	Trolox	
150	94.1 ± 0.2 **	^a^ IC_50_ = 46.7 ± 2.6 *** μg/mL	8.8	92.6 ± 0.2	^a^ IC_50_ = 2.9 ± 0.1 μg/mL
75	72.4 ± 0.7 *		6.2	83.6 ± 1.9	
37.5	35.0 ± 3.7 ***		3.8	49.6 ± 2.6	
18.75	15.1 ± 1.3 **		1.3	18.6 ± 0.4	
9.37	9.2 ± 0.1 ^ns^		0.6	9.9 ± 0.3	

^a^ IC_50_: The half-maximal inhibitory concentration. The findings are represented as mean ± standard deviation. ^ns^ *p* = 0.5; * *p* = 0.05; ** *p* = 0.01; *** *p* = 0.0001 show significant changes in comparison to trolox. The *T. purpurea* subsp. *apollinea* methanolic extract and trolox were compared using a *t*-test.

**Table 6:** Anticancer effects of *T. purpurea* subsp. *apollinea* methanol extract on cancer and non-cancer cell lines.

	^a^ IC_50_ (µg/mL)			
Cell Lines	*T. purpurea* subsp. *apollinea* Extract	95% Confidence Interval	R^2^ Value	Doxorubicin (Positive Control)
^b^ MCF7	102.9 ± 0.5 ***	93.8 to 113.5	0.9	37.4 ± 0.1
^c^ MG63	118.3 ± 2.4 ***	102.0 to 171.0	0.9	19.0 ± 0.3
^d^ T47D	114.7 ± 1.0 ***	103.0 to 135.0	0.9	7.0 ± 0.1
^e^ HeLa	196.2 ± 2.3 ***	171.1 to 211.2	0.9	41.9 ± 0.1
^f^ PC3	117.6 ± 1.0 ***	104.0 to 165.4	0.9	46.3 ± 0.2
^g^ U937	248.4 ± 7.5 ***	215.2 to 311.4	0.9	41.7 ± 0.9
^h^ WI38	242.9 ± 1.8 ***	207.5 to 374.8	0.9	20.1 ± 0.1

^a^ IC_50_: The half-maximal inhibitory concentration. ^b^ breast cancer (MCF7), ^c^ osteosarcoma (MG63), ^d^ breast ductal carcinoma (T47D), ^e^ cervical cancer (HeLa), ^f^ prostate cancer (PC3), ^g^ leukemia (U937) and ^h^ normal human fetal lung fibroblast (WI38). The findings are shown as mean ± standard deviation. *** *p* = 0.0001 show significant difference in comparison to doxorubicin. The *T. purpurea* subsp. *apollinea* and doxorubicin were compared using *t*-test analysis.

**Table 7:** Selectivity indices of *T. purpurea* subsp. *apollinea* methanol extract for cancer cell lines.

	^a^ SI					
Cell Lines	^b^ MCF7	^c^ MG63	^d^ T47D	^e^ HeLa	^f^ PC3	^g^ U937
*T. purpurea* subsp. *apollinea* Extract	2.3	2.0	2.1	1.2	2.0	0.9
Doxorubicin	0.5	1.0	2.8	0.4	0.4	0.4

^a^ SI: Selectivity index, ^b^ breast cancer (MCF7), ^c^ osteosarcoma (MG63), ^d^ breast ductal carcinoma (T47D), ^e^ cervical cancer (HeLa), ^f^ prostate cancer (PC3), ^g^ leukemia (U937).

## Data Availability

Not applicable.

## Sample Availability

Samples of the extracts are available from the authors.

## References

[1] Baskar R., Lee K.A., Yeo R., Yeoh K.-W. (2012). Cancer and radiation therapy: Current advances and future directions. Int. J. Med. Sci..

[2] Al-Saraireh Y.M., Alshammari F.O., Youssef A.M., Al-Sarayreh S., Almuhaisen G.H., Alnawaiseh N., Al Shuneigat J.M., Alrawashdeh H.M. (2021). Profiling of CYP4Z1 and CYP1B1 expression in bladder cancers. Sci. Rep..

[3] Yang C., Mai Z., Liu C., Yin S., Cai Y., Xia C. (2022). Natural products in preventing tumor drug resistance and related signaling pathways. Molecules.

[4] Al-Saraireh Y.M., Alshammari F.O., Youssef A.M., Al-Sarayra Y.M., Al-Saraireh R.A., Al-Muhaisen G.H., Al-Mahdy Y.S., Al-Kharabsheh A.M., Abufraijeh S.M., Alrawashdeh H.M. (2021). Cytochrome 4Z1 expression is correlated with poor prognosis in patients with cervical cancer. Curr. Oncol..

[5] Al-Saraireh Y.M., Alshammari F.O., Youssef A.M., Al-Sarayreh S., Al-Sarayra Y.M., Aborajooh E., Al-Shuneigat J., Alrawashdeh H.M. (2021). Screening of glypican-6 expression in benign, primary and metastatic colon cancers. Clin. Med. Insights Oncol..

[6] Alshammari F.O., Al-Saraireh Y.M., Youssef A.M., Al-Sarayra Y.M., Alrawashdeh H.M. (2021). Glypican-1 Overexpression in Different Types of Breast Cancers. Onco Targets Ther..

[7] Youssef A.M., Maaty D.A., Al-Saraireh Y.M. (2023). Phytochemical Analysis and Profiling of Antitumor Compounds of Leaves and Stems of *Calystegia silvatica* (Kit.) Griseb. Molecules.

[8] Forman H.J., Zhang H. (2021). Targeting oxidative stress in disease: Promise and limitations of antioxidant therapy. Nat. Rev. Drug Discov..

[9] Murphy M.P., Bayir H., Belousov V., Chang C.J., Davies K.J., Davies M.J., Dick T.P., Finkel T., Forman H.J., Janssen-Heininger Y. (2022). Guidelines for measuring reactive oxygen species and oxidative damage in cells and in vivo. Nat. Metab..

[10] Akbari B., Baghaei-Yazdi N., Bahmaie M., Mahdavi Abhari F. (2022). The role of plant-derived natural antioxidants in reduction of oxidative stress. BioFactors.

[11] Ayoka T.O., Ezema B.O., Eze C.N., Nnadi C.O. (2022). Antioxidants for the Prevention and Treatment of Non-communicable Diseases. J. Explor. Res. Pharmacol..

[12] Hussein R.M., Youssef A.M., Magharbeh M.K., Al-Dalaen S.M., Al-Jawabri N.A., Al-Nawaiseh T.N., Al-Jwanieh A., Al-Ani F.S. (2022). Protective Effect of Portulaca oleracea Extract Against Lipopolysaccharide-Induced Neuroinflammation, Memory Decline, and Oxidative Stress in Mice: Potential Role of miR-146a and miR-let 7. J. Med. Food.

[13] Boulos L. (1999). Flora of Egypt.

[14] Samuel V.J., Mahesh A.R., Murugan V. (2019). Phytochemical and pharmacological aspects of Tephrosia genus: A brief review. J. Appl. Pharm. Sci..

[15] Gulecha V., Sivakuma T. (2011). Anticancer activity of Tephrosia purpurea and Ficus religiosa using MCF 7 cell lines. Asian Pac. J. Trop. Med..

[16] Chen Y., Yan T., Gao C., Cao W., Huang R. (2014). Natural products from the genus Tephrosia. Molecules.

[17] Dahham S.S., Tabana Y.M., Iqbal M.A., Ahamed M.B., Ezzat M.O., Majid A.S., Majid A.M. (2015). The anticancer, antioxidant and antimicrobial properties of the sesquiterpene β-caryophyllene from the essential oil of Aquilaria crassna. Molecules.

[18] Fafal T., Tüzün B.S., KIVÇAK B. (2022). Fatty Acid Compositions and Antioxidant Activities of Ranunculus isthmicus subsp. tenuifolius and Ranunculus rumelicus. Int. J. Nat. Life Sci..

[19] Ibrahim O.H., Al-Qurashi A.D., Asiry K.A., Mousa M.A., Alhakamy N.A., Abo-Elyousr K.A. (2022). Investigation of Potential In Vitro Anticancer and Antimicrobial Activities of Balanites aegyptiaca (L.) Delile Fruit Extract and Its Phytochemical Components. Plants.

[20] Elsharkawy E.R., Alghanem S.M., Elmorsy E. (2021). Effect of habitat variations on the chemical composition, antioxidant, and antimicrobial activities of Achillea fragrantissima (Forssk) Sch. Bip. Biotechnol. Rep..

[21] Kumar P., Sati S., Khulbe K., Pant P., Tripathi A.N., Sarvendra K. (2022). Phytochemical constituents, antimicrobial and antioxidant activities of Kumaun Himalayan Hoop Pine bark extract. Nat. Prod. Res..

[22] Kumar R.S., Anburaj G., Subramanian A., Vasantha S., Selvam A.P. (2019). Preliminary phytochemical investigation, Antimicrobial activity and GC-MS analysis of leaf extract of Capparis zeylanica Linn. J. Pharm. Phytochem.

[23] Belakhdar G., Benjouad A., Abdennebi E. (2015). Determination of some bioactive chemical constituents from Thesium humile Vahl. J. Mater. Environ. Sci..

[24] Rakkimuthu R., Ananthi P., Sathishkumar P., Ananda kumar A.M., Sowmiya D. (2023). Chemical profiling of fern Cheilosoria mysurensis (Wall. ex Hook.) Ching & Shing and its biological activity. Plant Sci. Today.

[25] Mohamed N.T. (2021). Seperation of bioactive compounds from Haemolymph of scarab beetle Scarabaeus sacer (Coleoptera: Scarabaeidae) by GC-MS and determination of its antimicrobial activity. Int. J. Appl. Biol..

[26] Harun A., Aziz N.A., Azenan N.S.M., Kamarazzaman N.F.M., So’ad S.Z.M. (2020). Antimicrobial Efficacy, Antioxidant Profile and Nine Alternative Active Constituents from Petroleum Ether and Ethyl Acetate Extract of Entada spiralis. Malays. J. Anal. Sci..

[27] Hussein H.J., Hameed I.H., Hadi M.Y. (2017). Using gas chromatography-mass spectrometry (GC-MS) technique for analysis of bioactive compounds of methanolic leaves extract of Lepidium sativum. Res. J. Pharm. Technol..

[28] Rautela I., Dheer P., Thapliyal P., Joshi T., Sharma N., Sharma M.D. (2018). GC-MS analysis of plant leaf extract of Datura stramonium in different solvent system. Eur. J. Biomed. Pharm. Sci.

[29] Khan N., Ali A., Qadir A., Ali A., Warsi M.H., Tahir A., Ali A. (2021). GC-MS analysis and antioxidant activity of Wrightia tinctoria R. Br. leaf extract. J. AOAC Int..

[30] Abdelhamid M.S., Kondratenko E.I., Lomteva N.A. (2015). GC-MS analysis of phytocomponents in the ethanolic extract of Nelumbo nucifera seeds from Russia. J. Appl. Pharm. Sci..

[31] Morah F.N., Uduagwu D.N. (2017). Chemical composition, antioxidant and larvicidal activity of Alchornea laxiflora (Benth) leaf extracts. Edorium J. Pharmacol..

[32] Dawoud S.F., Al-Akra T., Zedan A.M. (2021). Antioxidant Activity of Some Natural Compounds in Alleviating the Hepatotoxicity Effects Induced by Emamectin Benzoate in Male Mice. J. Agric. Chem. Biotechnol..

[33] Emam K.K., Abdel Fattah M.E., El Rayes S.M., Hebishy M.A., Dessouki A.A. (2022). Assessment of Wheat Germ Oil Role in the Prevention of Induced Breast Cancer in Rats. ACS Omega.

[34] Ali H.A.-M., Mohammed Y.H., Imad H.H. (2016). Determination of metabolites products by Cassia angustifolia and evaluate antimicobial activity. J. Pharmacogn. Phytother..

[35] Chen Y.-F., Wu K.-J., Siao L.-R., Tsai H.-Y. (2022). Trilinolein, a Natural Triacylglycerol, Protects Cerebral Ischemia through Inhibition of Neuronal Apoptosis and Ameliorates Intimal Hyperplasia via Attenuation of Migration and Modulation of Matrix Metalloproteinase-2 and RAS/MEK/ERK Signaling Pathway in VSMCs. Int. J. Mol. Sci..

[36] Ould Bellahcen T., Cherki M., Sánchez J.A.C., Cherif A., El Amrani A. (2019). Chemical composition and antibacterial activity of the essential oil of Spirulina platensis from Morocco. J. Essent. Oil Bear. Plants.

[37] Varsha K.K., Devendra L., Shilpa G., Priya S., Pandey A., Nampoothiri K.M. (2015). 2, 4-Di-tert-butyl phenol as the antifungal, antioxidant bioactive purified from a newly isolated *Lactococcus* sp.. Int. J. Food Microbiol..

[38] Zhou C., Li C., Siva S., Cui H., Lin L. (2021). Chemical composition, antibacterial activity and study of the interaction mechanisms of the main compounds present in the Alpinia galanga rhizomes essential oil. Ind. Crops Prod..

[39] Gazwi H.S., Shoeib N.A., Mahmoud M.E., Soltan O.I., Hamed M.M., Ragab A.E. (2022). Phytochemical Profile of the Ethanol Extract of Malvaviscus arboreus Red Flower and Investigation of the Antioxidant, Antimicrobial, and Cytotoxic Activities. Antibiotics.

[40] Subash N., Raju G. (2014). GC-MS analysis and antibacterial activity of Stem of Indigofera longeracemosa Boiv. Ex Baill. Nat. Pharm. Technol..

[41] Rhetso T., Shubharani R., Roopa M., Sivaram V. (2020). Chemical constituents, antioxidant, and antimicrobial activity of Allium chinense G. Don. Future J. Pharm. Sci..

[42] Sunita A., Ganesh K., Sonam M. (2017). Screening and evaluation of bioactive components of Cenchrus ciliaris L. by GC-MS analysis. Int. Res. J. Pharm..

[43] Shariare M.H., Noor H.B., Khan J.H., Uddin J., Ahamad S.R., Altamimi M.A., Alanazi F.K., Kazi M. (2021). Liposomal drug delivery of Corchorus olitorius leaf extract containing phytol using design of experiment (DoE): In-vitro anticancer and in-vivo anti-inflammatory studies. Colloids Surf. B. Biointerfaces.

[44] Okpala E.O., Onocha P.A., Ali M.S. (2022). Antioxidant activity of phytol dominated stem bark and leaf essential oils of Celtis zenkeri Engl. Trends Phytochem. Res..

[45] Abdel-Hady H., El-Wakil E.A., Abdel-Gawad M. (2018). GC-MS analysis, antioxidant and cytotoxic activities of Mentha spicata. Eur. J. Med. Plants.

[46] Faridha Begum I., Mohankumar R., Jeevan M., Ramani K. (2016). GC–MS analysis of bio-active molecules derived from Paracoccus pantotrophus FMR19 and the antimicrobial activity against bacterial pathogens and MDROs. Indian J. Microbiol..

[47] Neveen M.K., Emad A.S., Dalia M.A., Enas M.A., Ahmed M.A.-E. (2014). Biological activities of secondary metabolites from Emericella nidulans EGCU 312. Afr. J. Microbiol. Res..

[48] Kotteswari M., Prabhu K., Rao M.R.K., Ahamed A., Balaji T., Dinakar S., Sundaram R.L. (2020). The gas chromatography-mass spectrometry study of one Ayurvedic formulation avipathi churnam. Drug Invent. Today.

[49] Zubair M.F., Ajibade S.O., Lawal A.Z., Yusuf S.A., Babalola J.B., Mukadam A.A., Hamid A.A. (2017). GC-MS analysis, Antioxidant and Antimicrobial Properties of Eclipta prostrata leaves. Int. J. Chem. Biochem. Sci..

[50] Radman S., Čižmek L., Babić S., Cikoš A.-M., Čož-Rakovac R., Jokić S., Jerković I. (2022). Bioprospecting of less-polar fractions of Ericaria crinita and Ericaria amentacea: Developmental Toxicity and antioxidant activity. Mar. Drugs.

[51] Anita A., Selvaraj D. (2022). In silico molecular docking study of plant-based compounds from medicinal plant Lantana camara L. against Aedes aegypti L. protein. Int. J. Mosq. Res..

[52] El-Sayed O.H., Asker M.M., Shash S.M., Hamed S.R. (2015). Isolation, structure elucidation and biological activity of Di-(2-ethylhexyl) phthalate produced by Penicillium janthinellum 62. Int. J. Chem. Tech. Res..

[53] Habib M.R., Karim M.R. (2012). Antitumour evaluation of di-(2-ethylhexyl) phthalate (DEHP) isolated from *Calotropis gigantea* L. flower/Evaluacija antitumorskog djelovanja di-(2-etilheksil)-ftalata (DEHP) izoliranog iz cvjetova *Calotropis gigantea* L.. Acta Pharm..

[54] Sirikhansaeng P., Tanee T., Sudmoon R., Chaveerach A. (2017). Major phytochemical as γ-sitosterol disclosing and toxicity testing in Lagerstroemia species. Evid.-Based Complement. Altern. Med..

[55] Jiang Y., Pei J., Zheng Y., Miao Y.-j., Duan B.-z., Huang L.-f. (2021). Gallic Acid: A Potential Anti-Cancer Agent. Chin. J. Integr. Med..

[56] Zhang X., Liu J., Qian C., Kan J., Jin C. (2019). Effect of grafting method on the physical property and antioxidant potential of chitosan film functionalized with gallic acid. Food Hydrocoll..

[57] Wang L., Pan X., Jiang L., Chu Y., Gao S., Jiang X., Zhang Y., Chen Y., Luo S., Peng C. (2022). The biological activity mechanism of chlorogenic acid and its applications in food industry: A review. Front. Nutr..

[58] Lee S.-H., Kim J.K., Kim D.W., Hwang H.S., Eum W.S., Park J., Han K.H., Oh J.S., Choi S.Y. (2013). Antitumor activity of methyl gallate by inhibition of focal adhesion formation and Akt phosphorylation in glioma cells. Biochim. Et Biophys. Acta (BBA)-Gen. Subj..

[59] Espíndola K.M.M., Ferreira R.G., Narvaez L.E.M., Silva Rosario A.C.R., Da Silva A.H.M., Silva A.G.B., Vieira A.P.O., Monteiro M.C. (2019). Chemical and pharmacological aspects of caffeic acid and its activity in hepatocarcinoma. Front. Oncol..

[60] Azaat A., Babojian G., Nizar I. (2022). Phytochemical Screening, Antioxidant and Anticancer Activities of Euphorbia hyssopifolia L. against MDA-MB-231 Breast Cancer Cell Line. J. Turk. Chem. Soc. Sect. A Chem..

[61] Gullón B., Lú-Chau T.A., Moreira M.T., Lema J.M., Eibes G. (2017). Rutin: A review on extraction, identification and purification methods, biological activities and approaches to enhance its bioavailability. Trends Food Sci. Technol..

[62] Sepúlveda L., Ascacio A., Rodríguez-Herrera R., Aguilera-Carbó A., Aguilar C.N. (2011). Ellagic acid: Biological properties and biotechnological development for production processes. Afr. J. Biotechnol..

[63] Pei K., Ou J., Huang J., Ou S. (2016). p-Coumaric acid and its conjugates: Dietary sources, pharmacokinetic properties and biological activities. J. Sci. Food Agric..

[64] Arya S.S., Rookes J.E., Cahill D.M., Lenka S.K. (2021). Vanillin: A review on the therapeutic prospects of a popular flavouring molecule. Adv. Tradit. Med..

[65] Kim J.K., Park S.U. (2019). A recent overview on the biological and pharmacological activities of ferulic acid. Excli J..

[66] Zhao Y., Liu S. (2021). Bioactivity of naringin and related mechanisms. Die Pharm.-Int. J. Pharm. Sci..

[67] Alshehri M.M., Sharifi-Rad J., Herrera-Bravo J., Jara E.L., Salazar L.A., Kregiel D., Uprety Y., Akram M., Iqbal M., Martorell M. (2021). Therapeutic potential of isoflavones with an emphasis on daidzein. Oxid. Med. Cell. Longev..

[68] David A.V.A., Arulmoli R., Parasuraman S. (2016). Overviews of biological importance of quercetin: A bioactive flavonoid. Pharm. Rev..

[69] Ruwizhi N., Aderibigbe B.A. (2020). Cinnamic acid derivatives and their biological efficacy. Int. J. Mol. Sci..

[70] Sen P., Sahu P.K., Haldar R., Sahu K., Prasad P., Roy A. (2016). Apigenin naturally occurring flavonoids: Occurrence and bioactivity. Pharm. Biosci. J..

[71] Bangar S.P., Chaudhary V., Sharma N., Bansal V., Ozogul F., Lorenzo J.M. (2022). Kaempferol: A flavonoid with wider biological activities and its applications. Crit. Rev. Food Sci. Nutr..

[72] Choi S.-S., Lee S.-H., Lee K.-A. (2022). A Comparative Study of Hesperetin, Hesperidin and Hesperidin Glucoside: Antioxidant, Anti-Inflammatory, and Antibacterial Activities In Vitro. Antioxidants.

[73] Ahmed Hassan L.E., Khadeer Ahamed M.B., Abdul Majid A.S., Iqbal M.A., Al Suede F.S.R., Haque R.A., Ismail Z., Ein O.C., Majid A.M.S.A. (2014). Crystal structure elucidation and anticancer studies of (-)-pseudosemiglabrin: A flavanone isolated from the aerial parts of Tephrosia apollinea. PLoS ONE.

[74] Azeez K.O., Shaker N.M., ElShamy M.M., Mogib M.A. (2015). Phytochemical and Biological Evaluation of Tephrosia apollinea. Res. J. Pharm. Biol. Chem. Sci..

[75] Hassan E.A., Adnan Iqbal M., S Dahham S., M Tabana Y., B Khadeer Ahamed M., MS Abdul Majid A. (2017). Colorectal, prostate and pancreas human cancers targeted bioassay-guided isolations and characterization of chemical constituents from Tephrosia apollinea. Anti-Cancer Agents Med. Chem. (Former. Curr. Med. Chem.-Anti-Cancer Agents).

[76] Cheruth A.J., Al Baloushi S.A., Karthishwaran K., Maqsood S., Kurup S.S., Sakkir S. (2017). Medicinally active principles analysis of Tephrosia apollinea (Delile) DC. growing in the United Arab Emirates. BMC Res. Notes.

[77] Rizvi T.S., Khan A.L., Ali L., Al-Mawali N., Mabood F., Hussain J., Adnan M., Al-Harrasi A. (2018). In vitro oxidative stress regulatory potential of Citrullus colocynthis and Tephrosia apollinea. Acta Pharm..

[78] Youssef A.M.M., EL-Swaify Z.A.S., Maaty D.A., Youssef M.M. (2021). Phytochemistry and Antiviral Properties of Two Lotus Species Growing in Egypt. Vitae.

[79] Youssef A.M.M., El-Swaify Z.A.S. (2018). Anti-Tumour Effect of two Persicaria species seeds on colon and prostate cancers. Biomed. Pharmacol. J..

[80] Youssef A., El-Swaify Z., Maaty D., Youssef M. (2020). Comparative study of two *Lotus* species: Phytochemistry, cytotoxicity and antioxidant capacity. J. Pharm. Pharmacogn. Res..

[81] Al-saraireh Y.M., Youssef A.M., Alshammari F.O., Al-Sarayreh S.A., Al-Shuneigat J.M., Alrawashdeh H.M., Mahgoub S.S. (2021). Phytochemical characterization and anti-cancer properties of extract of *Ephedra foeminea* (Ephedraceae) aerial parts. Trop. J. Pharm. Res..

[82] Al-Saraireh Y.M., Youssef A.M., Alsarayreh A., Al Hujran T.A., Al-Sarayreh S., Al-Shuneigat J.M., Alrawashdeh H.M. (2021). Phytochemical and anti-cancer properties of Euphorbia hierosolymitana Boiss. crude extracts. J. Pharm. Pharmacogn. Res..

